# Bioinformatic Analysis Revealed the Essential Regulatory Genes and Pathways of Early and Advanced Atherosclerotic Plaque in Humans

**DOI:** 10.3390/cells11243976

**Published:** 2022-12-08

**Authors:** Luling He, Andrea Palos-Jasso, Yao Yi, Manman Qin, Liang Qiu, Xiaofeng Yang, Yifeng Zhang, Jun Yu

**Affiliations:** 1 Key Laboratory for Pharmacology and Translational Research of Traditional Chinese Medicine of Nanchang, Centre for Translational Medicine, Jiangxi University of Chinese Medicine, Nanchang 330006, China; 2 Jiangxi Key Laboratory of Traditional Chinese Medicine for Prevention and Treatment of Vascular Remodeling Diseases, Nanchang 330006, China; 3 Department of Cardiovascular Sciences and Centre for Metabolic Disease Research, Lewis Katz School of Medicine, Temple University, Philadelphia, PA 19140, USA; 4 Institute of Gynecology and Obstetrics of traditional Chinese Medicine, Jiangxi University of Chinese Medicine, Nanchang 330006, China

**Keywords:** atherosclerosis, microarray data, bioinformatics, gene network, gene connectivity

## Abstract

Atherosclerosis (AS) is a lipid-induced, chronic inflammatory, autoimmune disease affecting multiple arteries. Although much effort has been put into AS research in the past decades, it is still the leading cause of death worldwide. The complex genetic network regulation underlying the pathogenesis of AS still needs further investigation to provide effective targeted therapy for AS. We performed a bioinformatic microarray data analysis at different atherosclerotic plaque stages from the Gene Expression Omnibus database with accession numbers GSE43292 and GSE28829. Using gene set enrichment analysis, we further confirmed the immune-related pathways that play an important role in the development of AS. We are reporting, for the first time, that the metabolism of the three branched-chain amino acids (BCAAs; leucine, isoleucine, and valine) and short-chain fatty acids (SCFA; propanoate, and butanoate) are involved in the progression of AS using microarray data of atherosclerotic plaque tissue. Immune and muscle system-related pathways were further confirmed as highly regulated pathways during the development of AS using gene expression pattern analysis. Furthermore, we also identified four modules mainly involved in histone modification, immune-related processes, macroautophagy, and B cell activation with modular differential connectivity in the dataset of GSE43292, and three modules related to immune-related processes, B cell activation, and nuclear division in the dataset of GSE28829 also display modular differential connectivity based on the weighted gene co-expression network analysis. Finally, we identified eight key genes related to the pathways of immune and muscle system function as potential therapeutic biomarkers to distinguish patients with early or advanced stages in AS, and two of the eight genes were validated using the gene expression dataset from gene-deficient mice. The results of the current study will improve our understanding of the molecular mechanisms in the progression of AS. The key genes and pathways identified could be potential biomarkers or new drug targets for AS management.

## 1. Introduction

Atherosclerosis (AS) is a chronic inflammatory disease of the arterial wall [1]. The primary lesion of AS is characterized by lipid deposition and accompanied by the proliferation of smooth muscle cells and fibrous matrix, which gradually form into atherosclerotic plaque [2]. Surface rupture of the plaque leads to cardiovascular and cerebrovascular diseases such as ischemic attack and stroke [3,4]. The pathogenesis of AS is mainly involved in endothelial dysfunction, abnormal smooth muscle cell (SMC) proliferation and migration, oxidized lipid deposition, vascular matrix changes, inflammatory cell infiltration, and oxidative stress [5,6]. These processes mainly involve immune cells, foam cells, vascular endothelial cells, and SMCs, and contribute to the formation of AS. Innate and adaptive immune responses that trigger inflammation have been identified in AS, and thus may be a target for developing a new therapy [7]. Vascular smooth muscle cells (VSMC) have also been confirmed to play an essential role in the development of AS. SMC transdifferentiation into macrophage-like and fibrochondrocyte-like cells has been demonstrated in AS [8,9]. Additionally, inhibition of VSMC phenotypic switching may be beneficial in the advanced stages of AS [10]. Although several studies have vastly improved our understanding of the pathogenesis of AS, AS is still the leading cause of death worldwide and poses heavy economic and social burdens in society [11]. The pathogenesis of AS still needs to be further explored to develop targeted treatment and early gene therapy.

Understanding the molecular and cellular processes that convert asymptomatic plaques into symptomatic ones may facilitate the development of preventive pharmacotherapy with unprecedented impact on cardiovascular mortality and morbidity. In recent years, a series of genome-wide associations have been performed to identify genetic loci for human cardiovascular diseases [12,13]. Microarray data have been widely used to measure the expression of genome-wide genes in relevant tissues and to identify genes and pathways associated with diseases such as heart disease [14], type II diabetes [15], and nonalcoholic fatty liver disease [16]. Microarray data studies in AS with relevant tissues have demonstrated that certain genes and pathways play a critical role in the progression of carotid atherosclerotic plaque [17,18]. Studies confirmed a central role for lipid accumulation, inflammation, and proteases in plaque instability, and highlighted hemoglobin metabolism and bone resorption as critical enriched pathways in plaques [19]. SMC-related functional categories were most significantly affected in plaques [20]. However, the differential gene co-expression network modules connectivity in different atherosclerotic plaque stages of AS and the key genes for immune-related pathways especially downregulated SMC-related pathways were studied poorly in vascular disease. In addition, the molecular mechanisms involved in the formation of atherosclerotic plaque have not been fully elucidated.

In this study, we performed bioinformatic analysis aiming to uncover the gene expression patterns of the development of AS in humans based on microarray data (GSE43292 and GSE28829). The key genes were identified by integrating PPI, GO and WGCNA analysis, and were validated based on the microarray data (GSE9083 and GSE168610) obtained from Gene Expression Omnibus (GEO) database. Firstly, we compared the gene expression of the non-plaque stage to the plaque stage and early stage to the advanced stage based on gene set enrichment analysis (GSEA) (Materials and methods 2.2) and gene expression pattern analysis (GEPA) (Materials and methods 2.3). Secondly, we constructed a weighted gene co-expression network to investigate differential co-expression network module connectivity in different atherosclerotic plaque stages. Then, we integrated the results of weighted gene co-expression network analysis (WGCNA), protein and protein interaction network (PPI), and go ontology (GO) analysis (Materials and methods 2.4–2.6) to reveal key genes that are related to immune and muscle system pathways during the development of AS. Finally, we performed the receiver operating characteristic (ROC) curves (Materials and methods 2.7) for key genes to classify early stage and advanced stage of AS and validated key genes using the gene expression dataset from murine samples with specific gene deficiencies. The analyses provide novel insight into the pathogenesis of atherosclerotic plaque and provide valuable information for developing new targets and drugs in AS.

## 2. Materials and Methods

### 2.1. Microarray Data Collection

Gene expression data from human carotid macroscopically intact tissue (non-plaque stage) and atheroma plaque (plaque stage) collected in 32 patients undergoing carotid endarterectomy were obtained from GSE43292, which contained 32 normal carotid artery samples and 32 corresponding atherosclerotic plaque samples [21]. The data were analyzed with Expression Console software (version 1.1, Stephen Fodor, San Francisco, CA, USA) using the default RMA summarization method. Furthermore, we also downloaded gene expression data of in early stage (*n* = 13) and advanced-stage (*n* = 16) atherosclerotic plaque from human wastid which were obtained from GSE28829 [22]. To validate key genes, we also downloaded the gene expression dataset of *TYROBP* gene (DAP12) deficient mouse brain from GSE9083, and RNAseq data on mouse hearts for *PLN* gene-deficient mice from GSE168610 [23].

### 2.2. Gene Set Enrichment and Differentially Expressed Genes Analysis

Gene set enrichment analysis (GSEA) was performed using GSEA software (version 4.1.0, Vamsi K Mootha, Cambridge, MA, USA) based on c5.bp.v7.0.symbols.gmt (GOBP) and c2.cp.kegg.v7.0 symbols.gmt (KEGG) reference gene sets that were downloaded from the official GSEA [24,25]. Briefly, GSEA is a computational method that determines whether a priori defined set of genes show statistically significant, concordant differences between two biological states. The number of permutations was set to 1000, and the permutation type was set as “gene set”. The nominal *p* value < 0.05 is the significance threshold of the gene set in GSEA analysis. Moreover, differentially expressed genes (DEGs) between two groups were identified using the DESeq2 package in R. DEGs were defined as those with adjusted *p* values < 0.05 (adjusted by the Benjamini Hochberg method) and fold change > 2.

### 2.3. Gene Expression Patterns Analysis

Gene expression patterns analysis (GEPA) was used to calculate the major regulated pathways in the development of AS using R software (version 4.2.0, Ross Ihaka and Robert Gentleman, Vienna, Austria ). First, we selected the top 500 most significantly upregulated and downregulated genes (adjusted *p* value < 0.05) from the comparison of non-plaque stage with plaque stage and early with advanced stage based on the datasets of GSE43292 and GSE28829. Next, we obtained common significantly upregulated and downregulated genes from selected top 500 differentially expressed genes. Finally, these common upregulated and downregulated genes from non-plaque to plaque stage and early stage to advanced stage were retained for subsequent analysis. The enrichment of GO and KEGG pathways was performed using R package “clusterProfile” in R.

### 2.4. Constructing Weighted Co-expression Gene Networks

Weighted gene co-expression networks were constructed and analyzed using the WGCNA [26] (Weighted Gene Co-expression Network Analysis) package in R, which calculated topological overlap measures among genes and assigned the genes into different modules through hierarchical clustering. A dynamic tree cutoff of 0.25 was set to merge similar trees. Module eigengene was also calculated using WGCNA, which is the first principal component of gene expression values in each module. Genes with more than 25% variance were used to construct the weighted gene co-expression network. Finally, a weighted gene co-expression network was constructed using 14,256 and 16,220 genes in GSE43292 and GSE28829 datasets, respectively. The enrichment of genes in each module was analyzed using the R package “clusterProfile” [27]. Visualization of the network was performed using Cytoscape (version 3.8.2, Paul Shannon, Washington, DC, USA) [28].

### 2.5. GO and KEGG Enrichment Analyses

The R package “clusterProfile” was used to perform Gene Ontology (GO) and Kyoto Encyclopedia of Genes and genomes (KEGG) pathway enrichment analysis [27]. The Benjamin–Hochberg approach was used to correct multiple tests. The adjusted *p* value ≤ 0.05 was used as a threshold of significance for the enriched GO and KEGG terms for target genes.

### 2.6. Protein-Protein Interaction Network Analysis

The search tool for retrieving interacting genes (STRING; https://www.string-db.org, accessed on 10 June 2022) is a database of known and predicted protein-protein interactions that can be used to predict and track the protein–protein interactions network. Analyzing the interaction between different proteins can provide new insight into the mechanism of AS. This study used the STRING database to construct the PPI network of common upregulated and downregulated genes in different atherosclerotic plaque stages.

### 2.7. Logistic Regression Models with the ROC Curve

The logistic regression model was constructed using glm in R. The key genes were identified as predictive variables, and the sample type with early stage or advanced stage was considered a binary responsive variable. The 3-fold cross-validation was performed to validate the accuracy of the logistic regression models by *caret* package in R. The receiver operating characteristic (ROC) curves were generated to evaluate the sensitivity and specificity of the logistic regression models. The average area under the curve (AUC) was calculated to assess the models’ accuracy.

## 3. Results

### 3.1. Identification of Key Gene Sets in Different Stages of Atherosclerotic Plaque

**Figure 1:**
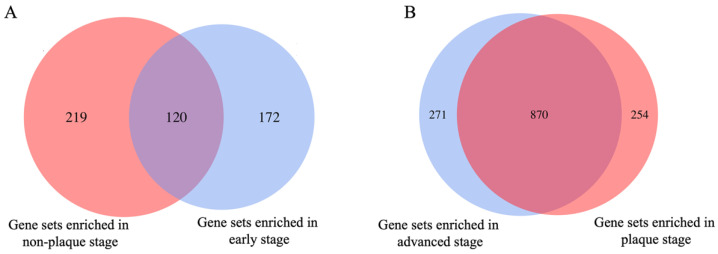
The summary of GSEA analysis based on GOBP and KEGG gene sets. (**A**) Venn diagram of gene sets enriched in non-plaque and early stage. (**B**) Venn diagram of gene sets enriched in plaque and advanced stage. The red color represents gene sets enriched in the non-plaque and plaque stages based on the dataset of GSE43292. The blue color represents gene sets enriched in the early stage and advanced stage based on the dataset of GSE28829.

To explore the key gene sets in different stages of atherosclerotic plaque, we performed the gene set enrichment analysis (GSEA) based on GOBP and KEGG gene sets, respectively. Using a *p* value threshold of 0.05, a total of 339 and 1124 gene sets were significantly enriched in non-plaque and plaque stages, respectively, when comparing non-plaque and plaque stages. Similarly, a total of 292 and 1141 gene sets were significantly enriched in early and advanced stages, respectively, when comparing early and advanced stages (Figure 1). Among these, 120 common gene sets were identified between gene sets enriched in non-plaque stage and early stage, and 870 common gene sets were identified between gene sets enriched in plaque stage and advanced stage (Figure 1). Notably, an average of 52% significantly enriched gene sets was common from non-plaque stage to plaque stage and early stage to the advanced stage of AS (Figure 1). This indicates the potential roles of these gene sets driving the progression of AS. To better comprehend which regulated gene sets play a more critical role in AS development, the top 10 GOBP and 10 KEGG gene sets enriched in different stages of atherosclerotic plaque were retained for further analysis. We found eight common enriched gene sets in non-plaque stage and early stage and 11 common enriched gene sets in plaque stage and advanced stage based on intersection of the top 10 GOBP and 10 KEGG enriched gene sets in corresponding stages. (Figure S1). For GOBP gene sets, myofibril assembly and cell communication by electrical coupling gene sets were significantly enriched in the non-plaque stage and early stage when compared to the non-plaque stage with plaque stage and early stage with advanced stage. Compared with the corresponding non-plaque stage and early stage, the gene sets, including adaptive immune response, leukocyte migration, positive regulation of leukocyte cell–cell adhesion and positive regulation of cell activation gene sets were significantly enriched in the plaque stage and advanced stage (Table 1). For KEGG gene sets, propanoate/butanoate metabolism, cardiomyopathy and valine, leucine and isoleucine degradation gene sets were significantly enriched in non-plaque stage and early stage when compared non-plaque stage with plaque stage and early stage with advanced stage. Compared with the corresponding non-plaque stage and early stage, cytokine–cytokine receptor interaction, intestinal immune network for IGA production, B cell receptor signaling pathway and Toll-like receptor signaling pathway gene sets were significantly enriched in the plaque and advanced stages of AS (Table 1). These results indicated that apart from the immune-related gene sets, the metabolism of short-chain fatty acids and branched-chain amino acids also plays a key role in different stages of atherosclerotic plaque.

### 3.2. The Mainly Regulated Pathways in Different Stages of Atherosclerotic Plaque

**Figure 2:**
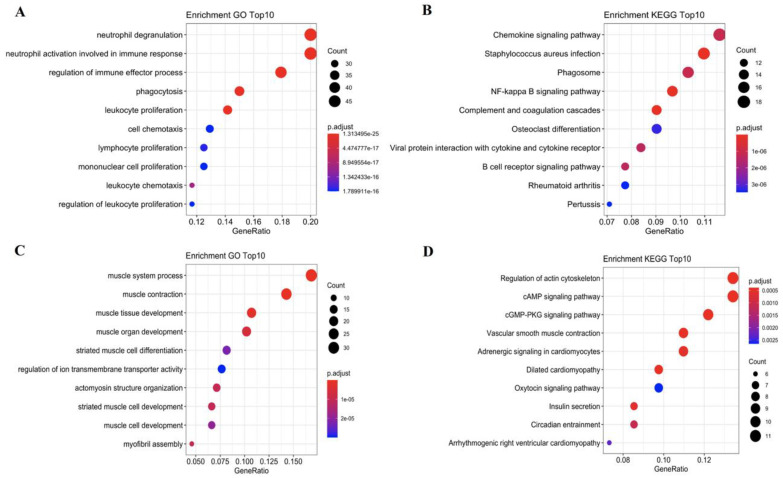
The functional enrichment analysis of common upregulated and downregulated genes using GO and KEGG analysis. (**A**,**B**) The top 10 enrichment pathways of 259 common upregulated genes using GO and KEGG analysis, respectively. (**C**,**D**) The top 10 enrichment pathways of 218 common downregulated genes using GO and KEGG analysis, respectively. The sizes of the dots represent the counts of enriched genes, and the dot color represents the adjusted *p*-value.

To investigate the mainly regulated pathways in different stages of atherosclerotic plaque, we selected the top 500 most significantly upregulated and downregulated genes from non-plaque to plaque in the dataset of GSE43292 and early to advanced stage in the dataset of GSE28829 (Table S1). Of them, 259 genes were significantly upregulated and 218 genes were significantly downregulated from non-plaque to plaque and early to advanced stage (Figure S2). Then, we performed GO and KEGG analysis for these commonly regulated genes in different stages of atherosclerotic plaque. The 259 common upregulated genes significantly enriched in 586 GOBP and 31KEGG signaling pathways (Table S2). In addition, the 218 common downregulated genes significantly enriched in 96 GOBP and 13 KEGG signaling pathways (Table S3). Interestingly, we found that these 259 common upregulated genes were mainly involved in B cell receptor and NF-kappa B signaling pathways to regulate immune-related biological processes, such as neutrophil activation in immune response, T cell activation, leukocyte cell–cell adhesion and mononuclear cell proliferation, etc (Figure 2A,B; Table S2). We also found that these 218 common downregulated genes were mainly enriched in biological processes, including muscle system process, muscle contraction and regulation of ion transmembrane transport, etc (Figure 2C; Table S3). KEGG analysis of the 218 common downregulated genes identified some pathways mainly associated with cAMP/cGMP-PKG signaling pathway and VSMC contraction (Figure 2D; Table S3). The results identified these immune and muscle related pathways as major regulated pathways in different stages of atherosclerotic plaque.

### 3.3. Remodeling of the Molecular Interaction Structure in Atherosclerosis

**Figure 3:**
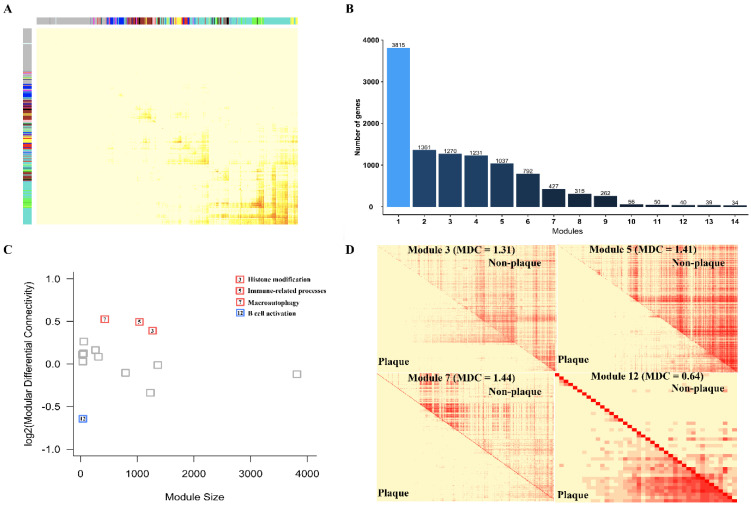
Differential modular connectivity analyses for individual genes in non-plaque and plaque stages. (**A**) Gene co-expression network module in different stages of atherosclerotic plaque. The rows and columns represent the same set of 14,256 genes with the first 75% variance in the dataset of GSE43292. (**B**) The numbers of modules and genes in weighted gene co-expression network modules based on the dataset of GSE43292. (**C**) Differential connectivity modules are highlighted. The y−axis represents the log2 transformed modular differential connectivity values. The x−axis denotes the number of genes in the corresponding modules. (**D**) Heatmap of gene-gene correlation in the differential connectivity of modules in the non-plaque stage (the upper right triangle of each module) compared with that in the plaque stage (the lower left triangle of each module).

**Figure 4:**
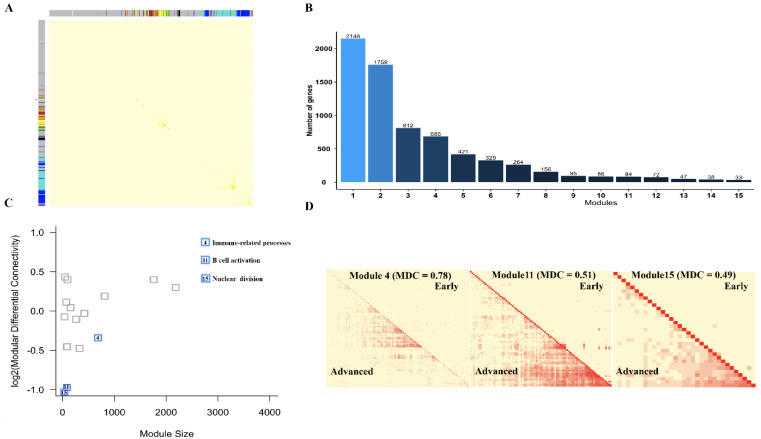
Differential modular connectivity analyses for early and advanced stage individual genes. (**A**) Gene co-expression network module in different stages of atherosclerotic plaque. The rows and columns represent the same set of 16,220 genes with the first 75% variance in the dataset of GSE28829. (**B**) The numbers of modules and genes in weighted gene co-expression network modules based on the dataset of GSE28829. (**C**) Differential connectivity modules are highlighted. The y-axis represents the log2 transformed modular differential connectivity values. The x-axis denotes the number of genes in the corresponding modules. (**D**) Heatmap of gene-gene correlation in the differential connectivity of modules in the early stage (the upper right triangle of each module) compared with that in the advanced stage (the lower left triangle of each module).

The number of neighboring genes associated with a gene in a network was defined as the connectivity of a gene which is an important index to identify the functional importance of a gene [29]. To characterize and compare the connectivity of genes in non-plaque stage and plaque stage, we constructed a multiple stage-weighted gene co-expression network encompassing 14,256 genes with the first 75% variance in 64 individuals and identified 14 network modules containing 34 to 3815 gene members (Figure 3A,B). Next, we used a metric known as modular differential connectivity (MDC) [30], which is defined as MDC = $\bar{\left|\mathrm{rA}\right|}$/$\bar{\left|\mathrm{rB}\right|}$, where $\bar{\left|\mathrm{rA}\right|}$ and $\bar{\left|\mathrm{rB}\right|\;}$ indicate the average absolute correlation coefficients of all pairwise gene members of a module in non-plaque stage and plaque stage, respectively. Using an empirical *p* value of 0.05 determined by a 1000 permutation test that shuffles the sample labels. Interestingly, we found that four modules were significantly differences in the co-regulation of genes in non-plaque stage and plaque stage (Figure 3C). Four modules are mainly related to histone modification (*p* = 1.0 × 10^−10^, module 3), T cell activation (*p* = 2.7 × 10^−24^, module 5), macroautophagy (*p* = 7.0 × 10^−3^, module 7) and B cell activation (*p* = 8.5 × 10^−6^, module 12) (Figure 3D; Tables S4 and S5). In addition, we also constructed a multiple stage-weighted gene co-expression network encompassing 16,220 genes with the first 75% variance in 29 individuals and identified 15 network modules containing 33 to 2148 gene members (Figure 4A,B). We also found that three modules related to immune-related processes (*p* = 9.8 × 10^−41^, module 4), B cell activation (*p* = 4.9 × 10^−10^, module 11) and nuclear division (*p* = 2.5 × 10^−15^, module 15) showed significantly diverse differential connectivity in the co-regulation of genes in early and advanced stages using MDC analysis (Figure 4C,D; Tables S6 and S7). Notably, genes with the function of immune-related processes, including macrophage, T cell and B cell activation clustered in modules, displayed significantly modular differential connectivity from non-plaque stage to plaque stage and early stage to advanced stage, respectively (Figure S3). In addition, the genes with the function of nuclear division only showed differential module connectivity in early stage and advanced stage. These results suggest that the connectivity of genes involved in these pathways exhibits large differences in the co-regulation of genes during the development of atherosclerotic plaque.

### 3.4. Identification of Key Genes As Potential Therapeutic targets for Atherosclerosis

**Figure 5:**
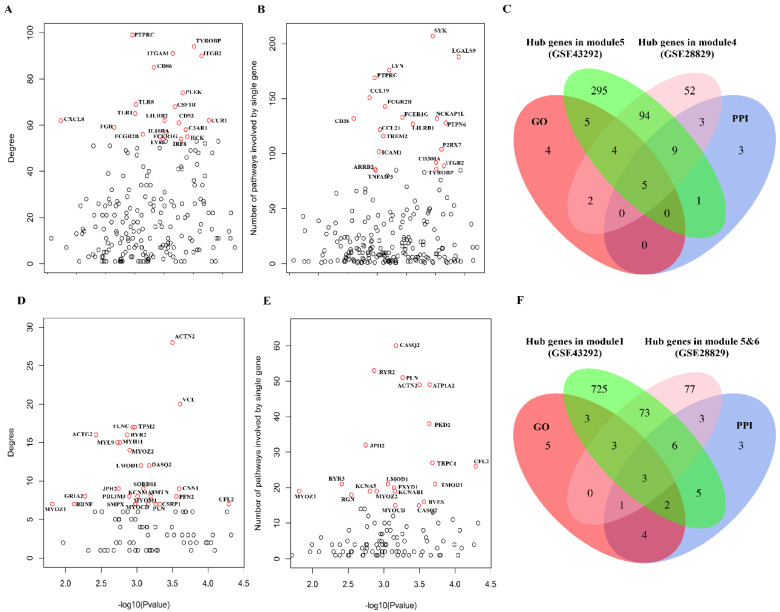
The eight essential genes were identified by integrating the results of PPI, GO, and Gene co-expression module analysis. (**A**) The top 20 genes with the highest degree value of protein-protein network interactions for 259 highly upregulated genes. (**B**) GO analysis demonstrates that the top 20 genes primarily involve immune-related pathways. (**C**) Five genes were identified as key genes for immune responses based on PPI, GO, and Gene co-expression module analysis results. (**D**) The top 20 genes with the highest degree value of protein-–protein network interactions for 218 highly downregulated genes. (**E**) The top 20 genes are involved in muscle-related pathways, as shown by GO analysis. (**F**) The results of three genes were identified as key for muscle system-related pathways based on PPI, GO, and Gene co-expression module analysis. Hub genes in modules represent genes exhibiting an absolute correlation coefficient > 0.85 within the eigengene of the modules related to immune or muscle-related pathways based on WGCNA analysis of GSE43292 and GSE28829 datasets. The dot represents different genes. The red dot represents the top 20 genes in the corresponding analysis.

**Figure 6:**
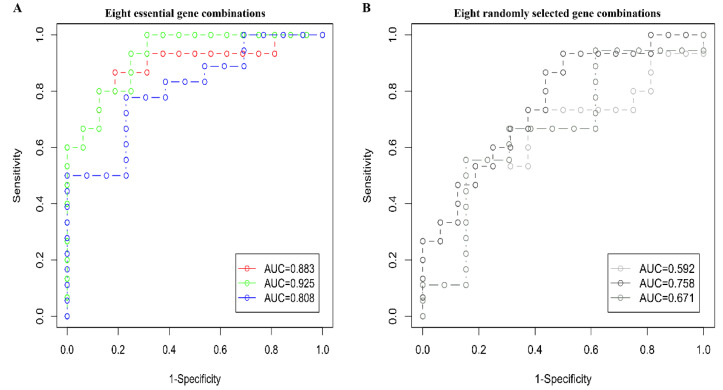
Receiver operating characteristic curves of eight essential genes and randomly selected eight genes. (**A**) The ROC curve of eight essential gene combinations is based on the logistic regression model. (**B**) The ROC curve of eight randomly selected gene combinations is based on the logistic regression model. The x-axis represented the 1-specificity of the negative–positive rate (false positive rate FPR), and the y-axis represented the true positive rate (TRR) sensitivity. Different colors represent the results of 3-fold cross-validation.

To reveal key genes related to the immune and muscle system in the development of atherosclerotic plaque, we integrated the results of GO, PPI, and WGCNA analysis. First, we examined the 259 and 218 common upregulated and downregulated genes in the context of network modules. The 259 common upregulated genes related to immune-related pathways are significantly enriched in module 5 (GSE43292) and module 4 (GSE28829) with modular differential connectivity in different stages of atherosclerotic plaque (Figure S4). The 218 common downregulated genes related to muscle system related pathways are significantly enriched in module 1 (GSE43292) and module 5 and 6 (GSE28829) (Figure S5). The enriched genes in module 5 and 6 (GSE28829) ed modular differential connectivity (Figure S5). These results further supported that these modules regulate the immune or muscle system related pathways in the development of atherosclerotic plaque. Next, we also performed the PPI analysis and calculated the number of immune-related pathways involved in a single gene based on the results of GO analysis for the 259 genes. The same analysis was performed in 218 genes to calculate the number of muscle system related pathways. Finally, we integrated the results of these three analyses and defined the genes with top 20 degree values in PPI network analysis, top 20 number of involved in the most immune or muscle-related pathways and exhibit an absolute correlation coefficient >0.85 within the eigengene of the enriched module as the key genes in the development of atherosclerotic plaque. Five genes (*PTPRC*, *FCGR2B*, *FCER1G*, *ITGB2* and *TYROBP*) were identified as key genes mainly involved in immune-related pathways, and *LMOD1*, *CFL2* and *PLN* genes were identified as key genes for muscle system related pathways in AS (Figure 5). These eight key genes were annotated using MGI and GWAS catalog databases (Table 2). We found that most of these key genes are involved in cardiovascular system phenotype from MGI database and lipid-related phenotype from GWAS catalog. Interestingly, we found that *LMOD1* is a target gene for coronary artery disease from GWAS catalog. Furthermore, we investigated the expression of key genes in different tissues following GTEx expression database (Figure S6). We found that these key genes were highly expressed in arteries, with *LMOD1* and *PLN* gene expression particularly higher when compared to other tissues (Figure S6). These results indicated that these key genes might play essential roles in developing atherosclerotic plaque. Finally, we used the 3-fold cross-validation method to evaluate the reliability of the model. The results showed that the AUC of the three verification sets in the logistic model constructed by the 3-fold cross-validation method was 0.883, 0.925 and 0.808 with an average AUC of 0.872 which much higher than the average of AUC of 0.674 with randomly selected eight genes (Figure 6). Taken together, these results suggest that these eight key genes could be used as therapeutic target genes in different stages of atherosclerotic plaque.

### 3.5. Validation of Potential Therapeutic Target Genes Using Gene Deficiency Mouse Expression Pro-Files

**Figure 7:**
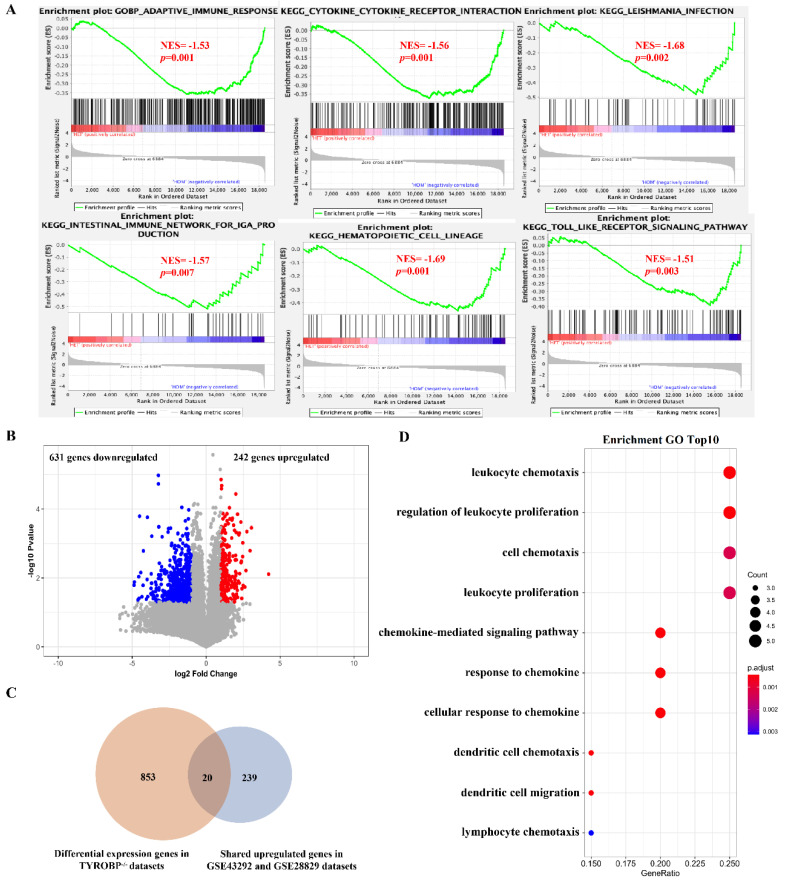
Validation of *TYROBP* key gene using gene expression profile of *TYROBP*^+/−^ and *TYROBP*^−/−^ mouse brain tissue. (**A**) GSEA analysis showed gene sets in *TYROBP*^−/−^ samples were significantly enriched. (**B**) Volcano diagram of the differentially expressed genes comparing samples from *TYROBP*^+/−^ and *TYROBP*^−/−^ mouse brain tissue. (**C**) Venn diagrams showing common genes between DEGs and commonly upregulated genes in GSE43292 and GSE28829 datasets. (**D**) The bubble diagram of GO-BP enrichment analyses of 20 common genes. Dot sizes represent counts of enriched common genes, and dot colors represent adjusted *p*-value.

**Figure 8:**
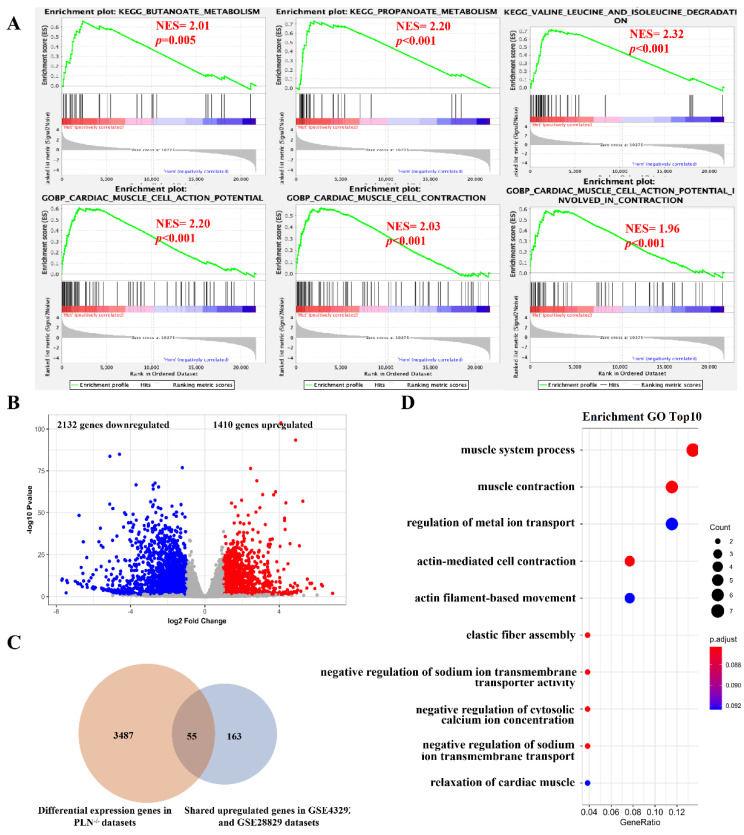
Validation of *PLN* key gene using gene expression profile of *PLN*^+/−^ and *PLN*^−/−^ mouse heart tissue. (**A**) Gene sets were significantly enriched in *PLN*^+/−^ samples based on GSEA analysis. (**B**) Representation of the differentially expressed genes comparing *PLN*^+/−^ and *PLN*^−/−^ mouse heart tissue. (**C**) Venn diagrams showing the common genes between DEGs and commonly downregulated genes in GSE43292 and GSE28829 datasets. (**D**) The bubble diagram of GO-BP enrichment analyses of 55 common genes. Dot sizes represent counts of enriched common genes, and dot colors represent adjusted *p*-value.

To validate the potential therapeutic target genes, we obtained the gene expression of *TYROBP* (DAP12) deficient mouse brain from the dataset of GSE9083, and RNAseq on mouse hearts from *PLN* deficient mice from the dataset of GSE168610. First, we performed gene set enrichment and differential gene expression analysis in these two datasets (Figure S7). For *TYROBP* gene dataset, most of the significantly enriched immune-related pathways in plaque and advanced stages are also significantly enriched in *TYROBP*-deficient mice (*TYROBP*^−/−^) in the comparison of *TYROBP*^−/−^ samples with *TYROBP*^+/−^ samples (Figure 7A). We found 873 significantly differentially expressed genes in comparing *TYROBP*^−/−^ samples with *TYROBP*^+/−^ samples (Figure 7B). Furthermore, among the 259 common upregulated genes, 20 genes with the functions related to immune pathways were also found in 873 significantly differentially expressed genes (Figure 7C,D). For *PLN* gene dataset, we also performed gene set enrichment and differential gene expression analysis between *PLN*^−/−^ samples and *PLN*^+/−^ samples (Figure S8). We found that the gene sets of butanoate/propanoate metabolism and valine/leucine/isoleucine degradation significantly enriched in non-plaque stage and early stage when compared non-plaque stage with the plaque stage early stage with advanced stage are also significantly enriched in *PLN*^+/−^ samples. Among the 218 gene-enriched pathways, cardiac muscle cell action potential and contraction pathways were also enriched in *PLN*^+/−^ samples (Figure 8A). In addition, we found that 3542 genes exhibited a significantly differential expression between *PLN*^−/−^ samples and *PLN^+/−^* samples (Figure 8B). Among the 218 common downregulated genes, 25% (55/218) of them with the function related to muscle system pathways also display significantly differential expression in the comparison of *PLN*^−/−^ samples and *PLN*^+/−^ samples (Figure 8C,D). These results further confirmed that *TYROBP* and *PLN* genes could regulate immune and muscle-related pathways to affect the development of atherosclerotic plaque.

## 4. Discussion

This study investigated the gene expression profiles of different atherosclerotic plaque stages with an accession number of GSE43292 and GSE28829. The key genes and pathways were identified based on systematic bioinformatic analysis. Two of eight key genes were validated using the gene expression dataset of gene-deficient murine samples, which are valuable for understanding the molecular mechanism in the development of atherosclerotic plaque.

Previous studies have used the microarray datasets of GSE43292 and GSE28829 to screen DEGs, key genes, and pathways in a single atherosclerotic plaque stage [17,18,31,32,33]. In comparison, the current study comprehensively investigated the gene expression profiles of atherosclerotic plaque in the stages of non-plaque to plaque as well as early to advanced. We report for the first time the differential modular connectivity in different stages of atherosclerotic plaque based on differential modular connectivity (MDC) analysis. We also performed gene set enrichment analysis (GSEA) in two databases, which can provide complementary information for differential gene expression analysis. Compared to the previous studies that identified key genes using PPI analysis [34,35], the current study established key genes by integrating the results of protein and protein interaction network (PPI), go ontology (GO), and modular differential connectivity (MDC) analysis in two datasets. The common regulated pathways and key genes were identified using the bioinformatic analysis of two datasets, which can effectively reduce the impact of confounding factors on the results of our study. We also validated two of eight key genes using the gene expression dataset of deficiency genes in mice.

Using gene set enrichment analysis with GOBP gene sets, we found that adaptive immune response, leukocyte migration, positive regulation of leukocyte cell-cell adhesion and positive regulation of cell activation gene sets were significantly enriched in the stages of plaque and advanced when compared to the corresponding stages of non-plaque and early in AS. These results indicated that the adaptive immune response and immune cell activation were turned on during the development of atherosclerotic plaque. Previous studies have shown that immune cells, such as B and T cells, contribute to AS [36,37] by playing an important role in the immune response and inflammation [17,38]. For KEGG analysis, we found that cytokine-cytokine receptor interaction, intestinal immune network for IGA production, B cell receptor signaling pathway, and Toll-like receptor signaling pathway gene sets were significantly enriched in the early stage of plaque and advanced lesions when compared to the corresponding stage of non-plaque and early plaque lesions, respectively. These results further supported that immune cells and immune-related pathways were activated in the development of atherosclerotic plaque. Interestingly, we also found that propanoate/butanoate metabolism and valine, leucine and isoleucine degradation gene sets were inhibited in the development of atherosclerotic plaque. It was previously reported that short-chain fatty acid (propanoate and butanoate) and branched-chain amino acid (valine, leucine, and isoleucine) are related to energy metabolism. For example, propionate and butyrate, the major metabolites of dietary fiber, are the main products of bacterial metabolism and constitute an essential source of energy [39]. The function of three branched-chain amino acids (BCAAs; leucine, isoleucine, and valine) can work together to modulate the insulin signal and glucose use by skeletal muscle [40]. Recent studies have suggested that the dysfunction of BCAA catabolism is associated with the risk of cardiovascular disease [41,42]. Therefore, the regulation of metabolism of short-chain fatty acids and branched-chain amino acids in the host may be a novel intervention strategy to hinder atherosclerotic plaque development.

Using gene pattern expression analysis, we investigated the highly regulated pathways in different stages of atherosclerotic plaque based on two datasets. The 259 commonly upregulated genes are mainly enriched in immune responses and immune cell activation during the development of atherosclerotic plaque. Immune-related signaling pathways, including B cell receptor and NF-kappa B, are classic signaling pathways widely reported in AS [37,43]. We also found 218 commonly downregulated genes that are typically involved in muscle system processes, muscle contraction, and regulation of ion transmembrane transport. Furthermore, cAMP/cGMP-PKG signaling pathway and VSMC contraction signaling pathways were enriched in the 218 commonly downregulated genes. VSMCs are the major cell types present in all stages of atherosclerotic plaque and play an essential role in the intervention of AS [44]. VSMC phenotypic switching affects plaque stability, and inhibiting phenotypic switching may benefit advanced AS [45], as seen by a reduction in atherosclerotic burden and improved fibrous cap stability when blocking [46]. Moreover, VSMC contraction mediated by ion transmembrane transport is rarely mentioned based on microarray data of different atherosclerotic stages. The intervention of ion transmembrane transport may be an effective method for treating and preventing hypertension and atherosclerosis. Previous studies also support that ion transmembrane transport [47].

Using modular differential connectivity analysis, we found that four modules displayed significant differential connectivity in non-plaque and plaque stages, and three modules displayed significant differential connectivity in early stage and advanced stage. The genes in modules 5 and 12 of multiple stages weighted gene co-expression network based on GSE43292 dataset and modules 4 and 11 of multiple stages weighted gene co-expression network based on GSE28829 dataset are mainly enriched in immune-related pathways including macrophage, T cell, and B cell activation, and these modules displayed modular differential connectivity. The genes associated with immune-related pathways identified by the GSEA and GEPA analyses also showed modular differential connectivity in corresponding modules by MDC analysis. These results further emphasized the important role of immune-related pathways in different stages of atherosclerotic plaque. This is consistent with the finding that innate as well as adaptive immune responses have been identified in AS, with components of T cell activation and antibody production during disease [7]. Our results demonstrated that modifying the co-regulation of immune-related genes in corresponding modules may interfere with atherosclerotic plaque development. We also found that the genes in module 7 of multiple stages weighted gene co-expression network based on GSE43292 dataset related to macroautophagy showed differential connectivity in the development of atherosclerotic plaque. The co-regulation of macroautophagy was stronger in non-plaque stage when compared with plaque stage. A previous study reported that the defective autophagy is one of the causes for atherosclerotic plaque [48]. The genes in module 15 of multiple stages weighted gene co-expression network based on GSE28829 dataset with the function of nuclear division showed higher co-regulation connectivity in advanced stage when compared to early stage, which indicates that nuclear division could be stronger in the advanced stage. Since uncontrolled nuclear division is a common feature of several human tumor cell lines [49], we speculated that the stronger co-regulation of genes associated with nuclear division in the advanced stage is one of the causes of the aneurysm. The regulation of co-expression of macroautophagy and nuclear division modules may be a new strategy for treating AS. Interestingly, we also found the biological pathway of B cell activation was enriched in modules 5 and 12 with differential connectivity based on multiple stages weighted gene co-expression network in GSE43292 dataset (Figure S9). Similarly, the pathway of B cell activation enriched in modules 4 and 11 with differential connectivity based on multiple stages weighted gene co-expression network in GSE28829 dataset (Figure S10). These results suggest that B cells have different functions in the development of atherosclerotic plaque. Our results further support that the B1 and B2 cells display unique functions in the development of AS [50]. The pathways of macroautophagy and nuclear division were not previously revealed by microarray data of different atherosclerotic stages, suggesting that MDC analysis provides new insights in the development of atherosclerotic plaque.

Immune and muscle system pathways play an important role in the development of atherosclerotic plaque. To uncover key genes related to immune and muscle system pathways in the development of atherosclerotic plaque, we integrated the results of GO, PPI and WGCNA analysis. *PTPRC*, *FCGR2B*, *FCER1G*, *ITGB2*, and *TYROBP* were identified as key genes regulating immune related pathways. *PTPRC* gene is an essential regulator of T and B cell antigen receptor-mediated activation [51], and its dysfunction can result in immunodeficiency, autoimmunity, or malignancy [52]. *FCGR2B* gene is mainly involved in a variety of effector and regulatory functions, such as phagocytosis of immune complexes and modulation of antibody production by B-cells [53]. The function of *FCER1G* gene is essential in chronic inflammation by correlating immune reactions [54]. *ITGB2* gene can mediate leukocyte migration through adhesive interactions between leukocytes and inflamed endothelial cells, which are critical for defense against bacteria and wound healing [55]. *TYROBP* gene is an adaptor in TREM2 signaling, and its activation can modulate cell proliferation, survival, and inflammation pathways [56]. Previous studies have reported TREM2 as a key player in Alzheimer’s disease [57] and *TYROBP* as key gene for AS [17,58]. Therefore, we speculate that AS and Alzheimer’s disease may have similar TREM2 signaling regulatory mechanisms. Likewise, *LMOD1*, *CFL2*, and *PLN* were identified as key genes in functions related to muscle processing systems. *LMOD1* gene has been identified as a causal gene for coronary artery disease by maintaining the differentiated SMC phenotype [59]. Mutations in *CFL2* gene mutations have been associated with congenital myopathies, including nemaline and myofibrillar myopathy [60]. *PLN* gene is an important regulator for sarcoendoplasmic reticulum (SR) calcium transport ATPase (SERCA), which uptakes Ca^2+^ to SR during the diastolic phase of cardiomyocytes to maintain intracellular calcium homeostasis [23]. Notably, *TYROBP* and *PLN* gene deficiency was validated in this study by analyzing gene expression in experimental mice. These genes will provide valuable information to understand the mechanisms underlying the progression of AS. The expression of key genes in multiple tissues based on GTEx expression database suggests that these key genes are highly expressed in arteries, with the expression of *LMOD1* and *PLN* genes being especially higher in arteries than in other tissues (Figure S6). The results further indicate that *LMOD1* and *PLN* genes may regulate the functional changes of VSMCs, thereby participating in the development of atherosclerotic plaque. Furthermore, we identified that the AUC of logistic regression model based on eight key gene combinations was 0.883, 0.925, and 0.808 with an average AUC of 0.872, much higher than the average AUC of 0.674 with randomly selected eight gene combinations. These results indicate that our logistic regression model for these key genes can reliably predict the stages of patients with AS.

The present study has several limitations. Firstly, compared to RNAseq, the microarray data might lead to some bias, which might affect the interpretation of the results. Secondly, there may be some confounding factors, including age and sex, which might affect gene expression, and could not be considered in this study. Thirdly, the key genes identified in the current study were not validated in animals in vivo or humans. Future studies using genetically modified animal and atherosclerotic animal models are warranted.

## 5. Conclusion

In summary, this study provides a comprehensive view of the molecular mechanisms at different stages of atherosclerotic plaque. It identifies several molecular mechanisms that potentially link the progression of atherosclerotic plaque. The differential modular connectivity in different stages of atherosclerotic plaque was first reported in this study. In addition, the eight key genes related to immune or muscle system pathways were considered to play a critical role in the development of atherosclerotic plaque. The eight key gene combinations can reliably predict the stages of patients with AS. Among these eight key genes, *TYROBP* and *PLN* genes were validated using the gene expression dataset of deficiency genes in mice. These results may help us better understand the functional mechanisms of AS in different atherosclerotic plaque stages. The essential genes and pathways found in this study might be potential biomarkers or drug targets for diagnosing or treating AS.

## Figures and Tables

**Table 1:** The gene set enrichment analysis of gene expression omnibus database with accession numbers GSE43292 and GSE28829.

GS	Types	*p* Value
Myofibril assembly	GOBP	<0.001 ^a^ and <0.001 ^b^
Cell communication by electrical coupling	GOBP	<0.001 ^a^ and 0.005 ^b^
Propanoate metabolism	KEGG	0.018 ^a^ and <0.001 ^b^
Butanoate metabolism	KEGG	0.011 ^a^ and <0.001 ^b^
Hypertrophic cardiomyopathy hcm	KEGG	<0.001 ^a^ and <0.001 ^b^
Dilated cardiomyopathy	KEGG	<0.001 ^a^ and <0.001 ^b^
Arrhythmogenic right ventricular cardiomyopathy arvc	KEGG	<0.001 ^a^ and 0.006 ^b^
Valine leucine and isoleucine degradation	KEGG	0.02 ^a^ and 0.006 ^b^
Adaptive immune response	GOBP	<0.001 ^c^ and <0.001 ^d^
Leukocyte migration	GOBP	<0.001 ^c^ and <0.001 ^d^
Positive regulation of leukocyte cell cell adhesion	GOBP	<0.001 ^c^ and <0.001 ^d^
Positive regulation of cell activation	GOBP	<0.001 ^c^ and <0.001 ^d^
Leukocyte cell cell adhesion	GOBP	<0.001 ^c^ and <0.001 ^d^
Cytokine cytokine receptor interaction	KEGG	<0.001 ^c^ and <0.001 ^d^
Leishmania infection	KEGG	<0.001 ^c^ and <0.001 ^d^
Intestinal immune network for iga production	KEGG	<0.001 ^c^ and <0.001 ^d^
B cell receptor signaling pathway	KEGG	<0.001 ^c^ and <0.001 ^d^
Hematopoietic cell lineage	KEGG	<0.001 ^c^ and <0.001 ^d^
Toll-like receptor signaling pathway	KEGG	<0.001 ^c^ and <0.001 ^d^

^a^ represents the *p* value of gene set significantly enriched at the stage of non-plaque stage; ^b^ represents the *p* value of gene set significantly enriched at the stage of early stage; ^c^ represents the *p* value of gene set significantly enriched at the stage of plaque stage; ^d^ represents the *p* value of gene set significantly enriched at the stage of advanced stage.

**Table 2:** The detailed annotation of key genes based on MGI and GWAS Catalog databases.

Genes	Full Name	Related Pathway (Gene Cards)	MGI Phenotype	Phenotypes from GWAS Catalog
PTPRC	Receptor-type tyrosine-protein phosphatase C	B Cell Receptor Signaling Pathway	cardiovascular system phenotype	low density lipoprotein cholesterol measurement
FCGR2B	Low affinity immunoglobulin gamma Fc region receptor II-b	B Cell Receptor Signaling Pathway	cardiovascular system phenotype	lipid measurement
FCER1G	High affinity immunoglobulin epsilon receptor subunit gamma	Innate Immune System	cardiovascular system phenotype	lipoprotein measurement
ITGB2	Integrin Subunit Beta 2	ERK Signaling	cardiovascular system phenotype	high density lipoprotein cholesterol measurement
TYROBP	TYRO protein tyrosine kinase-binding protein	Innate Immune System	immune system phenotype	NA
LMOD1	Leiomodin-1	Smooth Muscle Contraction	NA	coronary artery disease
CFL2	Cofilin-2	NA	muscle phenotype	low density lipoprotein cholesterol measurement
PLN	Phospholamban	Activation of cAMP-Dependent PKA	cardiovascular system phenotype	resting heart rate

## Data Availability

The datasets created and/or analyzed during the current study will be available from the corresponding author on reasonable request. There are no security, licensing, or ethical issues related to these data.

## Supplementary Materials

The following supporting information can be downloaded at: https://www.mdpi.com/article/10.3390/cells11243976/s1, Figure S1: Venn diagrams showing the common gene sets for the top 10 GOBP and 10 KEGG enriched gene sets in different atherosclerotic plaque stages based on the dataset of GSE43292 and GSE28829; Figure S2: The common genes for the top 500 most significantly differential expression genes in different atherosclerotic plaque stages based on the dataset of GSE43292 and GSE28829; Figure S3: The connectivity of genes related to macrophage cell activation in modules; Figure S4: The 259 common upregulated related to immune responses pathways in the co-expression modules; Figure S5: The 218 common downregulated related to muscle system pathways in the co-expression modules; Figure S6: The expression profiles of eight key genes in human tissues (from GTEx Portal: https://gtexportal.org/home/); Figure S7: The summary of GSEA analysis based on the dataset of GSE9083; Figure S8: The summary of GSEA analysis based on the dataset of GSE168610; Figure S9: The connectivity of genes related to B cell activation in module 5 and 12 based of multiple stages weighted gene co-expression network based on the dataset of GSE43292; Figure S10: The connectivity of genes related to B cell activation in module 4 and 11 based of multiple stages weighted gene co-expression network based on the dataset of GSE28829; Table S1: The differential genes with FDR < 0.01 in different stages of atherosclerotic plaques based on the dataset of GSE43282 and GSE28829; Table S2: The GO and KEGG analysis of the 259 common upregulated genes; Table S3: The GO and KEGG analysis of the 218 common downregulated genes; Table S4: The results of modular differential connectivity analysis based on dataset of non-plaque and plaque stage; Table S5: The enrichment pathways of module 3, 5, 7 and 12 using GO analysis; Table S6: The results of modular differential connectivity analysis based on dataset of early and advanced stage; Table S7: The enrichment pathways of module 4, 11 and 15 using GO analysis.
