# Supplementary material for: Bioinformatic Analysis Revealed the Essential Regulatory Genes and Pathways of Early and Advanced Atherosclerotic Plaque in Humans
Source: Cells. 2022 Dec 8;11(24):3976. doi: 10.3390/cells11243976 (PMC9776921; doi:10.3390/cells11243976)
Supplement: Supplementary file 1 [file cells-11-03976-s001.zip › cells-20274052_revised supplementary data_12_01.pdf]

Bioinformatic Analysis Revealed the Essential Regulatory Genes and Pathways of Early and Advanced Atherosclerotic Plaque in Humans

Luling He [1,2], Andrea Palos-Jasso [3], Yao Yi [4], Manman Qin [1,2], Liang Qiu [1,2], Xiaofeng Yang [3], Yifeng Zhang [1,2,*] and Jun Yu [3]

[1] Key Laboratory for Pharmacology and Translational Research of Traditional Chinese Medicine of Nan-chang, Centre for Translational Medicine, Jiangxi University of Chinese Medicine, Nanchang 330006, China

[2] Jiangxi Key Laboratory of Traditional Chinese Medicine for Prevention and Treatment of Vascular Re-modeling Diseases, Nanchang 330006, China

[3] Department of Cardiovascular Sciences and Centre for Metabolic Disease Research, Lewis Katz School of Medicine, Temple University, Philadelphia, PA 19140, USA

[4] Institute of Gynecology and Obstetrics of traditional Chinese Medicine, Jiangxi University of Chinese Medicine, Nanchang 330006, China

* Correspondence: zyf489662913@163.com

Supplementally Figures

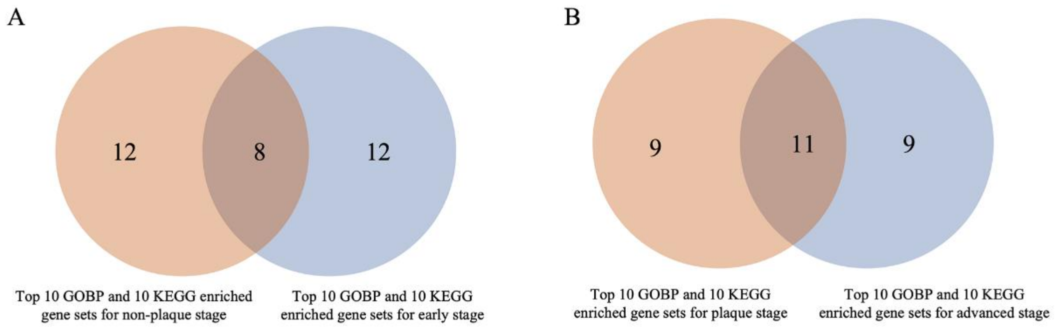

Figure S1 Venn diagrams showing the common gene sets for the top 10 GOBP and 10 KEGG enriched gene sets in different atherosclerotic plaque stages based on the dataset of GSE43292 and GSE28829. (A) Venn diagrams showing the common gene sets between the top 10 GOBP and 10 KEGG enriched gene sets in non-plaque stage and early stage when comparing non-plaque stage with plaque stage and early stage with the advanced stage. (B) Venn diagrams show the common gene sets between the top 10 GOBP and 10 KEGG enriched gene sets in plaque and advanced stages when compared to the non-plaque stage with plaque and early stage with advanced stage.

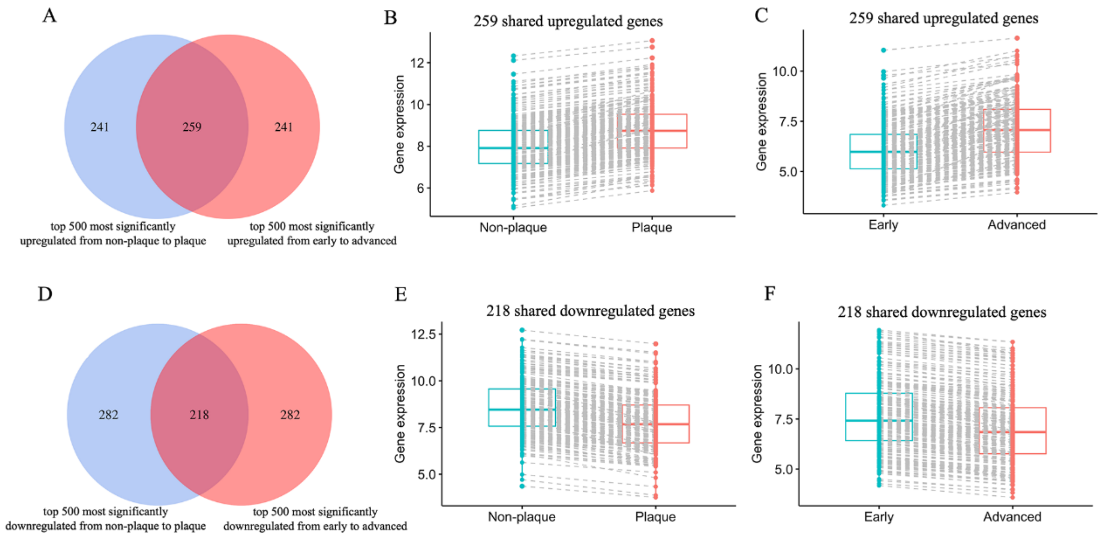

Figure S2 The common genes for the top 500 most significantly differential expression genes in different atherosclerotic plaque stages based on the dataset of GSE43292 and GSE28829. (A) The Venn diagram of the top 500 most significantly upregulated genes from non-plaque to plaque and early to the advanced stage. (B) The boxplot of 259 common upregulated genes from the non-plaque stage to the plaque stage in the dataset of GSE43292. (C) The boxplot of 259 common upregulated genes from early to advanced stage in the dataset of GSE28829. (D) The Venn diagram of the top 500 most significantly downregulated genes from non-plaque to plaque and early to advanced stage. (E) The boxplot of 218 common downregulated genes from non-plaque stage to the plaque stage in the dataset of GSE43292. (F) The boxplot of 218 common downregulated genes from early to advanced stage in the dataset of GSE28829.

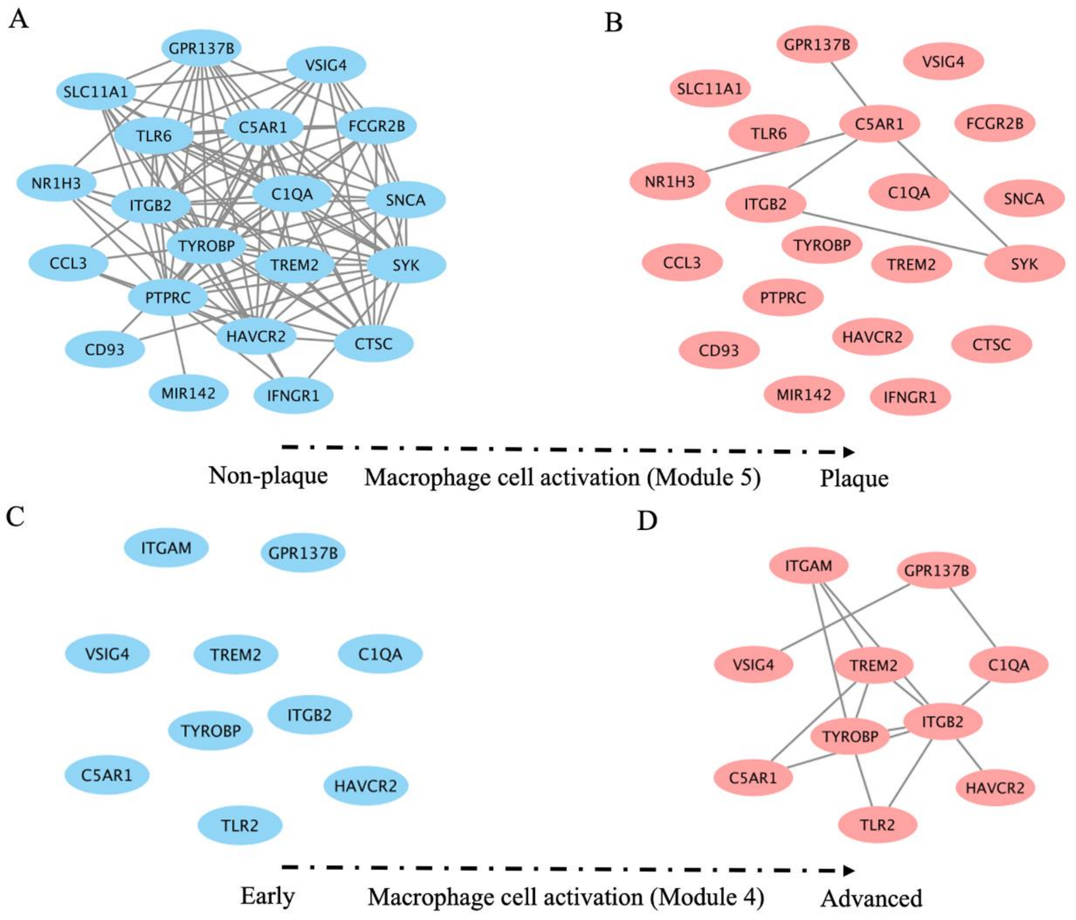

Figure S3 The connectivity of genes related to macrophage cell activation in modules. (A & B) The connectivity of genes related to macrophage cell activation in module 5 for the non-plaque stage and plaque stage based on multiple stages weighted gene co-expression network analysis in GSE43292. (C & D) The connectivity of genes related to macrophage cell activation in module 4 based on multiple stages weighted gene co-expression network analysis in GSE28829. Gene pairs with topological overlap matrix (TOM)>0.1 were connected with edges.

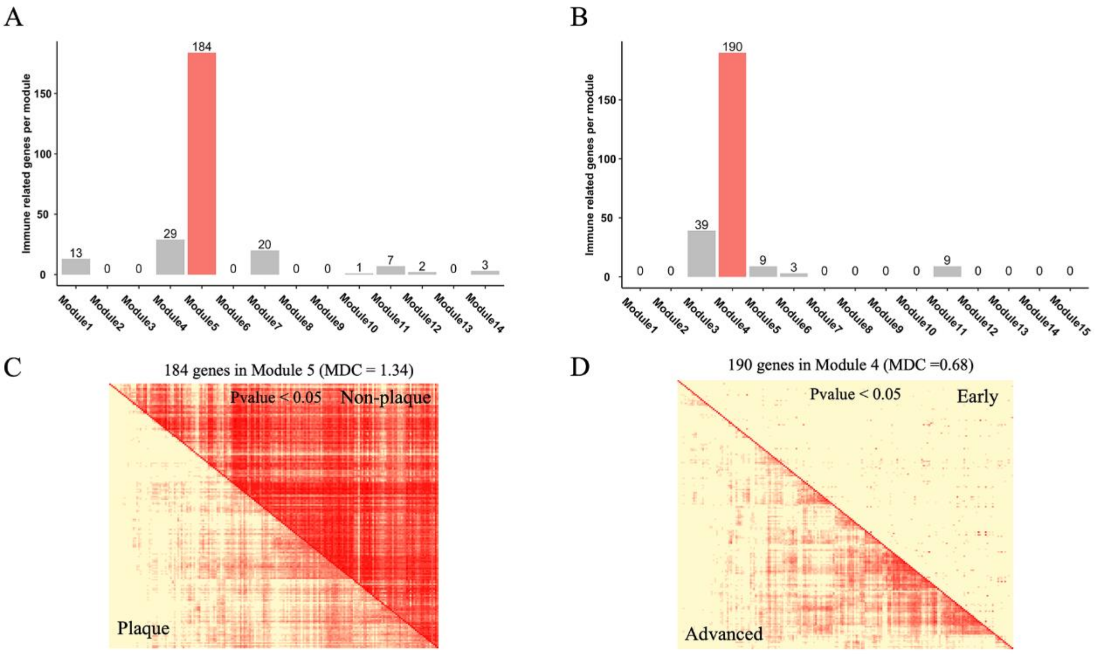

Figure S4 The 259 commonly upregulated genes related to immune response pathways in the co-expression modules. (A & B) Fold enrichment of the 259 commonly upregulated genes in the co-expression modules of multiple stages weighted gene co-expression network analysis in GSE43292 and GSE28829 datasets, respectively. (C) Heatmap of gene-gene correlation of genes enriched in module 5 of multiple stages weighted gene co-expression network based on the dataset of GSE43292. (D) Heatmap of gene-gene correlation of genes enriched in module 4 of multiple stages weighted gene co-expression network based on the dataset of GSE28829.

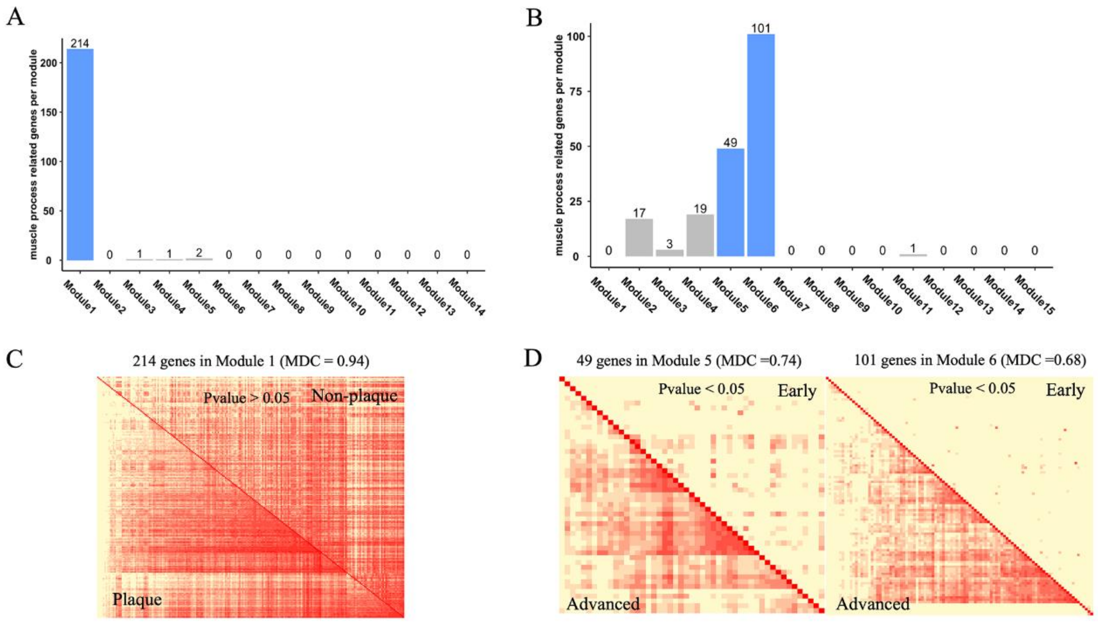

Figure S5 The 218 commonly downregulated genes related to muscle system pathways in the co-expression modules. (A & B) Fold enrichment of the 218 common downregulated genes in the co-expression modules multiple stages weighted gene co-expression network analysis in GSE43292 and GSE28829 datasets, respectively. (C) Heatmap of gene-gene correlation of genes enriched in module 1 of multiple stages weighted gene co-expression network based on the dataset of GSE43292. (D) Heatmap of gene-gene correlation of genes enriched in modules 5 and 6 of various stages weighted gene co-expression network based on the dataset of GSE28829.

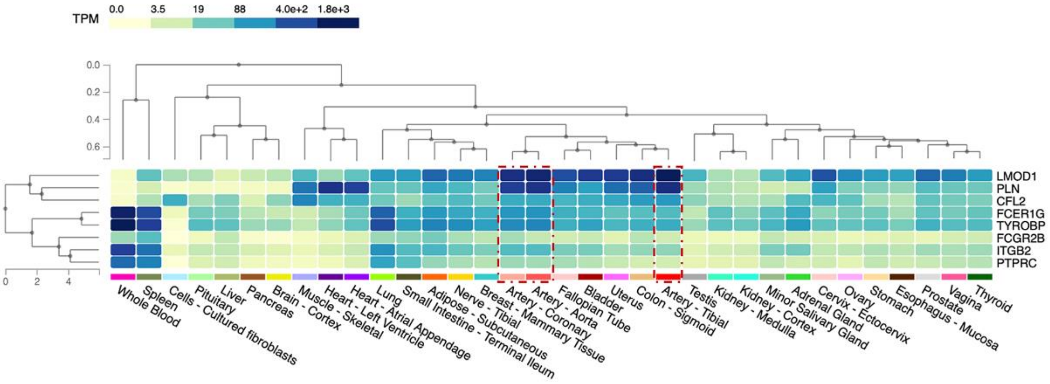

Figure S6 The expression profiles of eight critical genes in human tissues (from GTEx Portal: https://gtexportal.org/home/). The x-axis represents multiple tissues. Different colors represent the normalized gene expression.

A

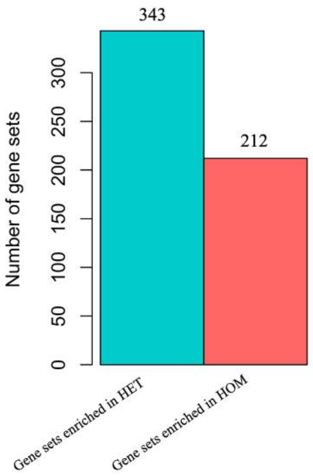

B

GeneSets	Groups	Types
Keratinization	GOBP	Gene sets enriched in TYROBP-/-mouse brain
Keratinocyte_differentiation	GOBP	Gene sets enriched in TYROBP-/-mouse brain
Antimicrobial_humoral_response	GOBP	Gene sets enriched in TYROBP-/-mouse brain
Cornification	GOBP	Gene sets enriched in TYROBP-/-mouse brain
Cytolysis	GOBP	Gene sets enriched in TYROBP-/-mouse brain
Reverse_cholesterol_transport	GOBP	Gene sets enriched in TYROBP-/-mouse brain
Myd88_dependent_toll_like_receptor_signaling_pathway	GOBP	Gene sets enriched in TYROBP-/-mouse brain
T_cell_mediated_immunity	GOBP	Gene sets enriched in TYROBP-/-mouse brain
Humoral_immune_response	GOBP	Gene sets enriched in TYROBP-/-mouse brain
Lymphocyte_mediated_immunity	GOBP	Gene sets enriched in TYROBP-/-mouse brain
Primary_immunodeficiency	KEGG	Gene sets enriched in TYROBP-/-mouse brain
Leukocyte_transendothelial_migration	KEGG	Gene sets enriched in TYROBP-/-mouse brain
Intestinal_immune_network_for_iga_production	KEGG	Gene sets enriched in TYROBP-/-mouse brain
Allograft_rejection	KEGG	Gene sets enriched in TYROBP-/-mouse brain
Systemic_lupus_erythematosus	KEGG	Gene sets enriched in TYROBP-/-mouse brain
Pathogenic_escherichia_coli_infection	KEGG	Gene sets enriched in TYROBP-/-mouse brain
Hematopoietic_cell_lineage	KEGG	Gene sets enriched in TYROBP-/-mouse brain
Leishmania_infection	KEGG	Gene sets enriched in TYROBP-/-mouse brain
Cytokine_cytokine_receptor_interaction	KEGG	Gene sets enriched in TYROBP-/-mouse brain
Alpha_linolenic_acid_metabolism	KEGG	Gene sets enriched in TYROBP-/-mouse brain

Figure S7 The summary of GSEA analysis based on the dataset of GSE9083. (A) Histogram of the number of genes sets enriched in TYROBP$^{+/-}$ and TYROBP$^{-/-}$ samples, respectively. (B) The top 10 enriched GOBP and KEGG gene sets in TYROBP$^{-/-}$ samples.

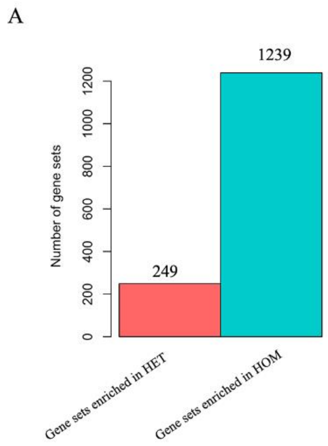

B

Genesets	Groups	Types
Mitochondrial respiratory chain complex assembly	GOBP	Gene sets enriched in PLN wt/R14del group
Respiratory electron transport chain	GOBP	Gene sets enriched in PLN wt/R14del group
Cellular respiration	GOBP	Gene sets enriched in PLN wt/R14del group
Atp synthesis coupled electron transport	GOBP	Gene sets enriched in PLN wt/R14del group
Mitochondrial gene expression	GOBP	Gene sets enriched in PLN wt/R14del group
Mitochondrial translation	GOBP	Gene sets enriched in PLN wt/R14del group
Nadh dehydrogenase complex assembly	GOBP	Gene sets enriched in PLN wt/R14del group
Oxidative phosphorylation	GOBP	Gene sets enriched in PLN wt/R14del group
Mitochondrial translational termination	GOBP	Gene sets enriched in PLN wt/R14del group
Mitochondrial electron transport nadh to ubiquinone	GOBP	Gene sets enriched in PLN wt/R14del group
Oxidative phosphorylation	KEGG	Gene sets enriched in PLN wt/R14del group
Citrate cycle tca cycle	KEGG	Gene sets enriched in PLN wt/R14del group
Parkinsons disease	KEGG	Gene sets enriched in PLN wt/R14del group
Valine leucine and isoleucine degradation	KEGG	Gene sets enriched in PLN wt/R14del group
Fatty acid metabolism	KEGG	Gene sets enriched in PLN wt/R14del group
Propanoate metabolism	KEGG	Gene sets enriched in PLN wt/R14del group
Peroxisome	KEGG	Gene sets enriched in PLN wt/R14del group
Huntingtons disease	KEGG	Gene sets enriched in PLN wt/R14del group
Aminoacyl trna biosynthesis	KEGG	Gene sets enriched in PLN wt/R14del group
Lysine degradation	KEGG	Gene sets enriched in PLN wt/R14del group

Figure S8 The summary of GSEA analysis based on the dataset of GSE168610.

(A) Histogram of the number of genes sets enriched in $PLN^{+/-}$ and $PLN^{-/-}$ samples, respectively. (B) The top 10 enriched GOBP and KEGG gene sets in $PLN^{+/-}$ samples.

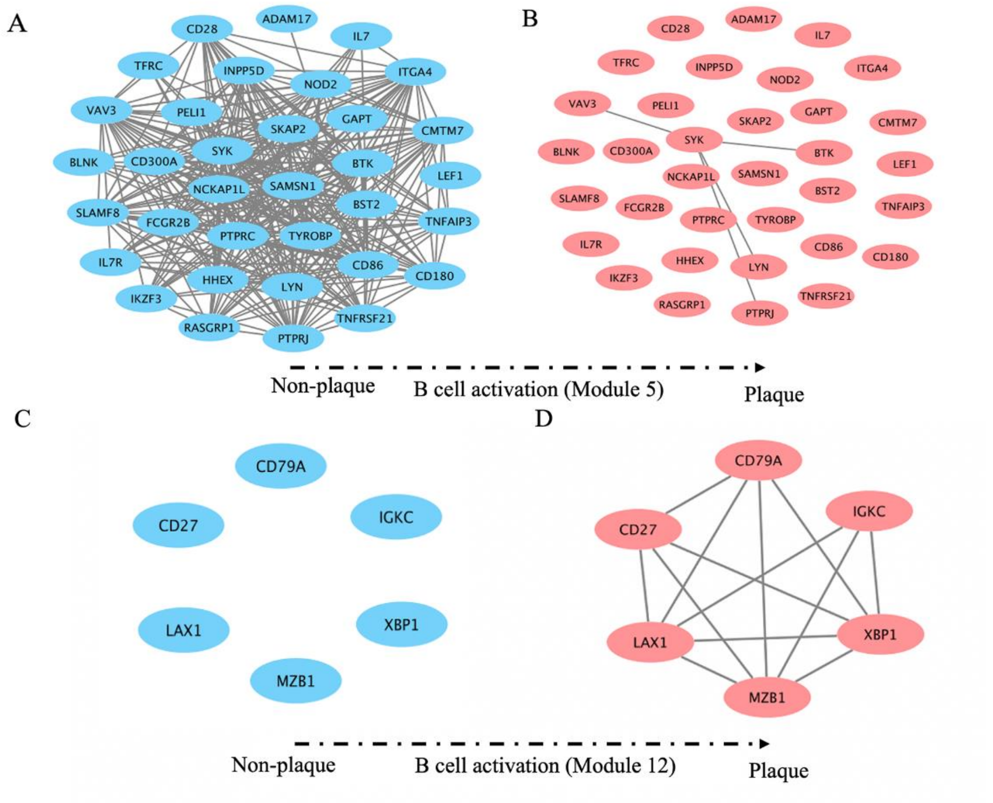

Figure S9 The connectivity of genes related to B cell activation in modules 5 and 12 based on multiple stages weighted gene co-expression network from the dataset of GSE43292. (A & B) The connectivity of genes related to B cell activation in module 5 for the non-plaque stage and plaque stage based on multiple stages weighted gene co-expression network analysis in GSE43292. (C & D) The connectivity of genes related to B cell activation in module 12 for the non-plaque stage and plaque stage based on multiple stages weighted gene co-expression network analysis in GSE43292. Gene pairs with topological overlap matrix (TOM)>0.1 were connected with edges.

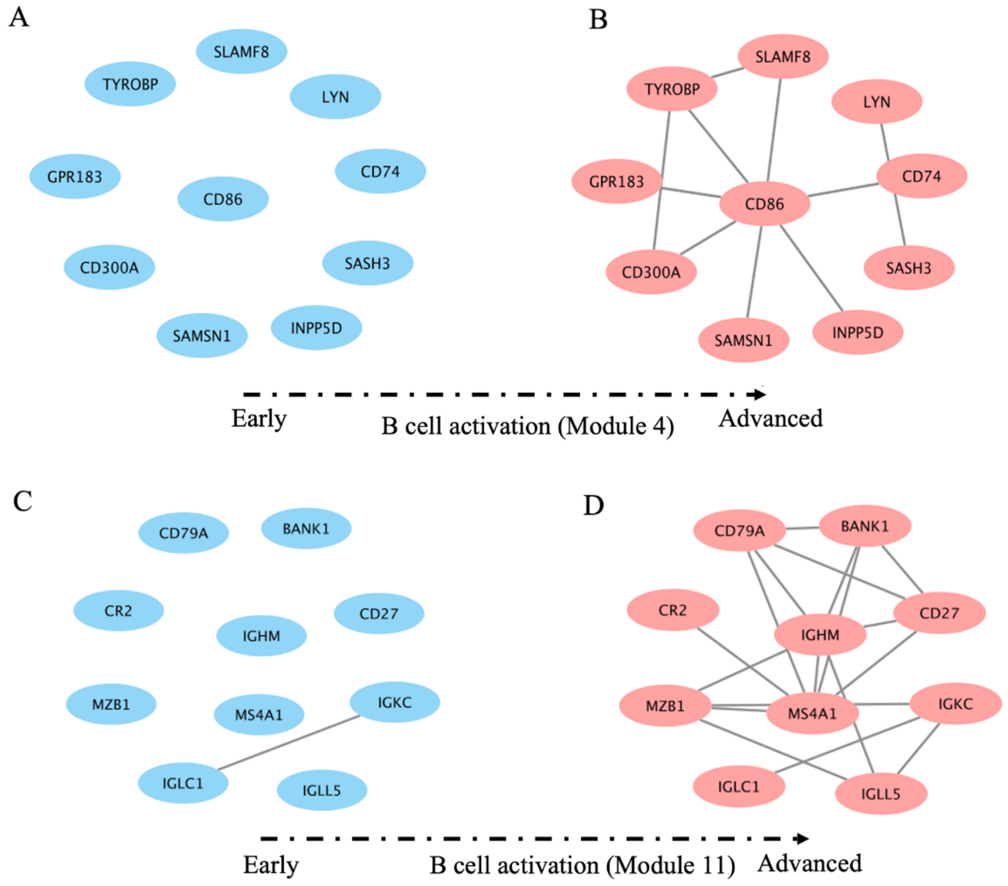

Figure S10 The connectivity of genes related to B cell activation in modules 4 and 11 based on multiple stages weighted gene co-expression network from the dataset of GSE28829. (A & B) The connectivity of genes related to B cell activation in module 4 for early and advanced stages based on multiple stages weighted gene co-expression network analysis in GSE28829. (C & D) The connectivity of genes related to B cell activation in module 11 for early and advanced stages based on multiple stages weighted gene co-expression network analysis in GSE28829. Gene pairs with topological overlap matrix (TOM)>0.1 were connected with edges.

Supplementally Tables

Table S1 The differential genes with FDR < 0.01 at different stages of atherosclerotic plaques based on the datasets of GSE43282 and GSE28829.

Table S2 The GO and KEGG analysis of the 259 commonly upregulated genes.

Table S3 The GO and KEGG analysis of the 218 commonly downregulated genes.

Table S4 The modular differential connectivity analysis results based on datasets of non-plaque and plaque stages.

Table S5 The enrichment pathways of modules 3, 5, 7, and 12 using GO analysis.

Table S6 The results of modular differential connectivity analysis based on datasets of early and advanced stages.

Table S7 The enrichment pathways of modules 4, 11, and 15 using GO analysis.
